# Genetic polymorphisms in monoamine neurotransmitter systems show only weak association with acute post-surgical pain in humans

**DOI:** 10.1186/1744-8069-2-24

**Published:** 2006-07-18

**Authors:** Hyungsuk Kim, Hyewon Lee, Janet Rowan, Jaime Brahim, Raymond A Dionne

**Affiliations:** 1 National Institute of Nursing Research, National Institutes of Health, Bethesda, MD, USA; 2 Pain and Neurosensory Mechanisms Branch, National Institute of Dentaland Craniofacial Research, National Institutes of Health, Bethesda, MD, USA; 3 Department of Nursing, Magnuson Clinical Research Center, National Institutes of Health, Bethesda, MD, USA; 4 Clinical Research Core, National Institute of Dental and CraniofacialResearch, National Institutes of Health, Bethesda, MD, USA

## Abstract

**Background:**

Candidate gene studies on the basis of biological hypotheses have been a practical approach to identify relevant genetic variation in complex traits. Based on previous reports and the roles in pain pathways, we have examined the effects of variations of loci in the genes of monoamine neurotransmitter systems including metabolizing enzymes, receptors and transporters on acute clinical pain responses in humans.

**Results:**

Variations in the catecholamine metabolizing enzyme genes (*MAOA *and *COMT*) showed significant associations with the maximum post-operative pain rating while the serotonin transporter gene (*SLC6A4*) showed association with the onset time of post-operative pain. Analgesic onset time after medication was significantly associated with the norepinephrine transporter gene (*SLC6A2*). However, the association between *COMT *genetic variation and pain sensitivity in our study differ from previous studies with small sample sizes, population stratification and pain phenotype derived from combining different types of pain stimuli. Correcting for multiple comparisons did not sustain these genetic associations between monoamine neurotransmitter systems and pain sensitivity even in this large and homogeneous sample.

**Conclusion:**

These results suggest that the previously reported associations between genetic polymorphisms in the monoamine neurotransmitter systems and the interindividual variability in pain responses cannot be replicated in a clinically relevant pain phenotype.

## Background

Considering moderate heritability estimates [1], multiple pain mechanisms [2] and complex networks of pain related molecules [3], individual variance in pain sensitivity arises from a complex network of multiple gene polymorphisms and environmental factors. The contribution of each gene is subtle on multiple mechanisms, making its signal difficult to detect. Therefore, evaluating millions of SNPs to find a few implicated in a phenotype like pain can be challenging. It is still necessary to choose candidate gene regions based on their biological role [4] when testing multiple SNPs to minimize the risk of false findings. Even if a polymorphism in a coding region does not result in an amino acid change, or if it is not in a coding sequence, it can still affect gene function by altering the stability, splicing or localization of the mRNA [5]. It is suggested that non-coding RNAs constitute a critical hidden layer of gene regulation in complex organisms [6].

Together with serotonin and histamine, catecholamine neurotransmitters such as dopamine, norepinephrine and epinephrine are collectively termed as monoamine neurotransmitters. Catecholamines and other monoamines operate through G protein-coupled receptors and second messenger systems to regulate the responsiveness of a large area of brain circuitry. They are synthesized in a highly restricted number of nuclei in the brain stem and basal forebrain, whose neurons project widely to targets in cortical and subcortical regions in the brain and in the spinal cord. Neural systems involved in higher brain functions such as emotion and cognition are affected by monoamine neurotransmitters. These molecules can be catabolized enzymatically; however, the functional activity of synaptically released monoamine neurotransmitters is primarily terminated by their reuptake into the nerve terminal [7].

Among the genes encoding molecules involved in monoamine neurotransmitter systems, the catechol O-methyltransferase gene (*COMT*) contains a common functional polymorphism, *COMT G*^1947^*A*, also known as *COMT Val*^158^*Met*, which substitutes from valine to methionine at amino acid position 158 (or 108 of S-COMT). This genetic variation is suggested to increase pain report and to decrease brain opioid system activation after an experimental pain challenge involving infusion of hypertonic saline into the masseter muscle [8]. Haplotypes including *COMT Val*^158^*Met *were recently identified and an association was suggested with experimental pain sensitivity and a chronic pain condition [9]. It is surprising that common variations in a single SNP or gene act dominantly on a complex behavior such as pain with hundreds of molecules involved in the composite phenotype.

Despite suggestive evidence that genetic polymorphisms in monoamine neurotransmitter related genes, including *COMT*, influence pain sensitivity, their exact roles in the perception, interpretation and behavioral expression of pain in humans are currently unknown. Rakvag et al[10], for example, reported that cancer patients with the *COMT Val/Val *genotype needed more morphine compared to the *Met/Met *genotype suggesting that the *Val/Met *polymorphism is not fully predictive of low tolerance to pain [8]. We have examined the effects of the variations in human genes encoding molecules involved in monoamine neurotransmitter systems including loci of *COMT *,monoamine oxidase A and B (*MAOA, MAOB*), norepinephrine transporter (*SLC6A2*), dopamine transporter (*SLC6A3*), serotonin transporter (*SLC6A4*) and dopamine receptor type 2 (*DRD2*) on acute clinical pain responses to investigate the contributions of genetic factors on pain sensitivity in humans.

## Results

The minor allelic frequencies of each SNP in *COMT, MAOA, MAOB, SLC6A2, SLC6A3, SLC6A4 *and *DRD2 *are shown in Table 1. They follow Hardy-Weinberg equilibrium except for SNP 12 of *COMT *and SNPs 1,2 and 3 of *DRD2*.

**Table 1:** Information of genotyped SNPs

Gene	Order	SNP ID	Location from transcription site	Nucleotide variation	Rarer allele frequency
*COMT*	1	rs5746846	-8,663	G/C	0.43
	2	rs2020917	-425	C/T	0.29
	3	rs933271	2,099	T/C	0.29
	4	rs5993882	8,225	T/G	0.25
	5	rs740603	15,869	G/A	0.43
	6	rs4646312	19,029	T/C	0.40
	7	rs165722	19,705	T/C	0.45
	8	rs6269	20,644	A/G	0.40
	9	rs4633	20,927	T/C	0.46
	10	rs4818	21,899	C/G	0.41
	11	rs4680	21,963	A/G	0.46
	12	rs174699	25,150	T/C	0.03
	13	rs165728	27,715	T/C	0.04
*MAOA*	1	rs3788862	1,956	A/G	0.34
	2	rs909525	37,794	T/C	0.38
	3	rs2283274	44,168	A/G	0.38
	4	rs1800659	58,761	A/G	0.33
	5	rs979606	85,734	T/C	0.33
	6	rs979605	85,955	G/A	0.33
	7		87,983	C/T	0.34
	8	rs2064070	93,274	T/A	0.36
*MAOB*	1	rs2283729	63,640	C/T	0.28
	2	rs3027452	83,893	C/T	0.20
	3	rs1799836	113,683	G/A	0.47
*SLC6A2*	1		7,551	C/T	0.06
	2	rs40434	8,968	A/G	0.41
	3	rs36017	28,261	G/C	0.49
*SLC6A3*	1	rs6350	2,347	C/T	0.09
	2	rs403636	7,192	G/T	0.14
	3	rs40184	50,469	G/A	0.41
	4	rs12516948	54,177	T/C	0.35
	5	rs10064219	60,548	C/T	0.14
*SLC6A4*	1	rs2066713	10,852	C/T	0.38
	2		22,674	G/A	0.01
	3	rs140701	24,184	G/A	0.45
	4	rs4325622	36,247	A/G	0.49
	5	rs1979572	50,741	C/T	0.48
*DRD2*	1	rs12421616	8,105	G/A	0.12
	2	rs4322431	12,929	A/T	0.26
	3	rs4581480	21,411	A/G	0.13
	4	rs4936272	26,977	G/A	0.46
	5	rs4648318	32,495	A/G	0.29
	6	rs11608185	51,187	G/A	0.49
	7	rs2734837	59,336	A/G	0.47
	8	rs6277	62,706	T/C	0.46
	9	rs6279	65,092	G/C	0.28

**Figure 1:**
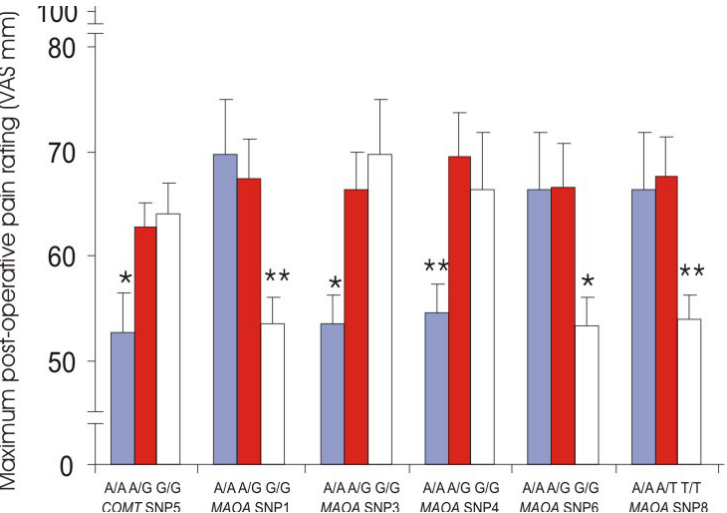
Maximum post-operative pain ratings and SNPs from *COMT *and *MAOA*. *COMT*: catechol O-methylatransferase gene *MAOA*: monoamine oxidase A gene, females only *, p < 0.05 **, p < 0.01

For clinically-induced acute pain, *COMT *SNP5 (rs740603) showed significant association with maximum post-operative pain rating. Homozygous A/A patients of SNP5 rated their maximum post-operative pain (N = 20, mean 52.6 mm; 95% CI, 44.5 to 60.6) lower (ANOVA, F = 3.34, df = 106, p = 0.039) than those of heterozygotes (N = 53, mean 62.8 mm; 95% CI, 58.4 to 67.2) and G/G homozygotes (N = 34, mean 63.9 mm; 95% CI, 57.5 to 64.5) (Figure 1).

Since the *MAOA *and *MAOB *are in the X chromosome and gender differences influence pain ratings [11-13], females and males were analyzed separately to investigate the association. Most SNPs of *MAOA *in females showed association with maximum post-operative pain ratings reflecting that the linkage disequilibrium of the genetic variations throughout the entire gene is high (see Additional file 1). Patients of homozygous G/G for *MAOA *SNP1 (rs3788862) reported maximum post-operative pain (N = 25, mean 53.5 mm; 95% CI, 48.2 to 58.5) lower (ANOVA, F = 5.82, df = 56, p = 0.005) than heterozygous (N = 24, 67.3 mm; 95% CI, 59.4 to 75.3) and A/A homozygous patients (N = 8, mean 69.6 mm; 95% CI, 57.2 to 82.1) (Figure 1). Homozygous *MAOA *SNP3 (rs2283724) A/A patients reported maximum post-operative pain (N = 23, mean 53.5 mm; 95% CI, 47.8 to 59.1) lower (ANOVA, F = 5.04, df = 56, p = 0.010) than heterozygous (N = 26, 66.3 mm; 95% CI, 58.8 to 73.8) and G/G homozygous patients (N = 8, mean 69.6 mm; 95% CI, 57.2 to 82.1). Homozygous SNP4 (rs1800659) A/A patients of *MAOA *reported maximum post-operative pain (N = 25, mean 54.6 mm; 95% CI, 49.2 to 59.9) lower (ANOVA, F = 5.11, df = 53, p = 0.009) than heterozygous (N = 21, 69.4 mm; 95% CI, 60.5 to 78.3) and G/G homozygous patients (N = 8, mean 66.4 mm; 95% CI, 53.9 to 78.8). Homozygous SNP6 (rs979605) G/G patients of *MAOA *reported maximum post-operative pain (N = 24, mean 53.3 mm; 95% CI, 48.0 to 58.7) lower (ANOVA, F = 4.63, df = 51, p = 0.014) than heterozygous (N = 20, 66.7 mm; 95% CI, 57.8 to 75.4) and A/A homozygous patients (N = 8, mean 66.4 mm; 95% CI, 53.9 to 78.8). Homozygous SNP8 (rs2064070) T/T patients of *MAOA *reported maximum post-operative pain (N = 25, mean 53.8 mm; 95% CI, 48.6 to 58.9) lower (ANOVA, F = 5.18, df = 55, p = 0.009) than heterozygous (N = 23, 67.5 mm; 95% CI, 59.6 to 75.4) and A/A homozygous patients (N = 8, mean 66.4 mm; 95% CI, 53.9 to 78.8) (Figure 1). When we analyzed the tag SNPs (SNP1, 2, 4 and 5) of *MAOA *together in females; the 2 most common combinations (G_T_A_T and A_C_G_C) represent 90.1% of the total existing 9 combinations. Though the homozygous patients with G_T_A_T/G_T_A_T (N = 17, 51.3 mm; 95% CI, 44.3 to 58.3) tended to report lower maximum post-operative pain than heterozygous (G_T_A_T/A_C_G_C, N = 14, 63.7 mm; 95% CI, 53.4 to 74.0) and homozygous (A_C_G_C/A_C_G_C) patients (N = 6, 64.7 mm; 95% CI, 50.3 to 79.1), this association was not statistically significant (p = 0.056). No significant association between clinical pain and *MAOA *genetic variations was found in male patients.

**Figure 2:**
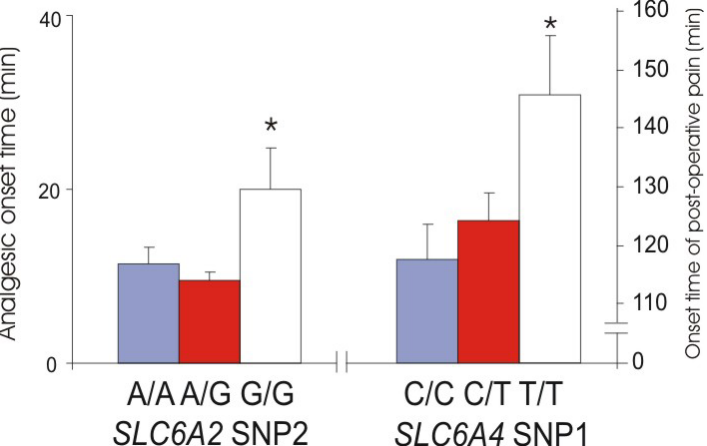
Time dependant pain responses and monoamine neurotransmitter transporter SNPs. *C6A2*: norepinephrine transporter gene *SLC6A4*: serotonin transporter gene *, p < 0.05

Homozygous SNP2 (rs40434) G/G patients of the norepinephrine transporter gene (*SLC6A2*) reported longer analgesic onset time (mean 20.2 mins; 95% CI, 9.7 to 30.6) after medication (ANOVA, F = 4.69, df = 93, p = 0.011) than heterozygous (9.5 mins; 95% CI, 7.8 to 11.2) and A/A homozygous patients (mean 11.3 mins; 95% CI, 7.3 to 15.3). Homozygous SNP1 (rs2066713) T/T patients of the serotonin transporter gene (*SLC6A4*) reported the onset of post-operative pain (mean 145.7 mins; 95% CI, 124.3 to 167.0) longer (ANOVA, F = 3.85, df = 101, p = 0.025) than heterozygous (124.4 mins; 95% CI, 115.4 to 133.5) and C/C homozygous patients (mean 117.6 mins; 95% CI, 105.2 to 130.0) (Figure 2).

## Discussion

It is well-known that allele frequencies of functional variants often differ substantially among groups that have different geographic ancestries [14]. Therefore, our results represent the association from only European Americans in our cohort. In order to generalize our findings, further studies with data collected from other major ethnic population groups are needed. The ethnicity of the subjects was self-determined instead of using ancestry informative markers. It is generally acceptable that use of self-identified ethnicity based on the geographic ancestry is an appropriate surrogate [15], although less reliable than using explicit genetic data [16]. Allelic frequencies of the genotyped SNPs showed consistent results with NCBI data, which suggest the generalizability of our sample to a European American population. Among the 5 ethnic clusters (sub-Saharan Africans; Europeans and Asians west of the Himalayas; East Asians; inhabitants of New Guinea and Melanesia; and Native Americans) based on 377 genetic markers [17], sub-Saharan Africans and East Asians are other major ethnic groups that need to be investigated.

The mis-sense SNP 11 (rs4680) of *COMT *markedly reduces enzyme activity to about 20–40% of wild-type levels [18-20]. This amino acid change may regulate the amounts of active dopamine and norepinephrine in various parts of the brain and therefore may be associated with mood and other mental processes which are closely related to pain perception [21-23] as well as the pathophysiology of neuropsychiatric disease [20,24]. However, it did not affect clinically induced acute pain responses in our sample. This is not consistent with previous findings reporting higher responses of met/met homozygotes at 158 amino acid position [8]. SNP 11 met/met homozygotes at codon 158, claiming to be associated with higher pain sensitivity, showed no significant association individually with clinically induced acute pain responses in our sample. An opposite trend was suggested as the met/met subjects showed lower pain responses than val/met and val/val subjects though it was not statistically significant (data not shown). Other studies also failed to replicate the higher pain responses in met/met homozygotes [10,25]. This discrepancy may be explained by the small sample size of Zubieta et al. which compared a val/val homozygous group (n = 3) to a heterozygous group (n = 11), and the met/met homozygous group (n = 4) to a heterozygous subgroup (n = 9 of the 11). The authors did not provide ethnic information of the subjects; even a small amount of population admixture can undermine an association study and lead to false positive results [26]. Differences in the pain induced experimental stimuli (saline injection into the masseter muscle) verses surgical extraction of impacted teeth and subsequent acute inflammation may also produce different phenotypes that activate different pathways.

Inconsistency was also found in the analysis of haploblocks. In contrast to previously reported lower sensory ratings of pain in G_C_G_G haplotypes [9], our haplotype data (not shown) demonstrated that G_C_G_G homozygotes are actually included in the higher pain sensitive populations, though the association is not very strong. Diatchenko et al. reported association between *COMT *variations and experimental pain and chronic pain conditions based on a sample composed of 85% European Americans and 15% from other ethnic populations. The experimental pain phenotype combined pressure pain thresholds, thermal pain thresholds and tolerance, temporal summation of thermal pain, ischemic pain threshold and tolerance. These different stimulus modalities likely cause pain via different neural mechanisms. Thermal and cold pain sensitivity, for example, are genetically dissociable [11] and there are at least 5 fundamentally different types of nociception [27], which may be inappropriate to mix for genetic analyses. The association between *COMT *and risk of developing temporomandibular disorders in Diatchenko et al. is based on 15 TMD patients out of 170 subjects and may be confounded by the ethnic admixture. Analyses for the individual SNP8 (rs6269) and SNP10 (rs4818) also did not show any significant association in our patient sample while Diatchenko et al. reported significant associations (p < 0.01). Thus, our failure to replicate previous studies related to *COMT *and pain [8,9] may be due to the small sample sizes, population stratification and the composite pain phenotype used in the latter publications.

Instead of SNP 11, SNP 5 (rs740603) in intron 1 showed moderate but significant association with maximum post-operative pain rating. The observed relationship between SNP5 of *COMT *and maximum post-operative pain rating suggests that another mechanism including different, unidentified but significant genetic factors may play a role in acute clinical pain perception. Even if a polymorphism is not in a coding sequence, it can still affect gene function by altering the stability, splicing, localization of mRNA or generating a small RNA. Emerging evidence suggests that the non-coding portions (i.e., introns) of the protein coding gene transcripts play an important role in regulatory pathways. Regulatory elements for gene expression in introns [28,29] or intron-derived microRNAs [30] are good examples. DNA sequences might be involved in the three-dimensional positioning of chromosomes in the nucleus enabling chromosome-chromosome interactions. Any portion of genomic DNA can be meaningful for the phenotype because of the complicated dynamics of DNA structure and gene expression [31]. Gene expression is generally regulated by DNA regions outside of gene regions that actually encode proteins, and there is much that is not yet understood about this process [32]. The information in the genome sequence must be considered in the large context of the chromosome [33] as well as in relationship with encoding proteins.

MAO, another catabolizing enzyme of monoamine neurotransmitters, is present in two isoforms (MAO A and MAO B), which share 70% amino acid sequence identity. These two forms differ with regard to several biochemical properties including their substrate specificity, cellular localization and regulation by pharmacologic agents. They are encoded by two closely linked genes with 15 exons on the X chromosome organized in opposite directions, tail to tail, 24 kb apart. However, MAO A and MAO B genes (*MAOA *and *MAOB*) have different tissue specific expression and functions which may be caused by differences in gene regulation including TATA box and Sp1 site [34]. While compulsive/aggressive behavior was observed in *MAOA *knockout mice [35], *MAO *activity has not been investigated for its possible association with pain phenotypes. The association studies between genetic polymorphisms of *MAOA*, *MAOB *and other human behaviors show inconsistent results [36-38].

We analyzed females and males separately for *MAOA *and *MAOB *because they are located on the X chromosome. Generally, males tend to report pain stimuli lower than females and they only have one allele of homozygotes. Therefore, heterozygous patients who can only be females, may show false higher pain ratings than homozygotes who are mixed females and males. It is not surprising that most SNPs from *MAOA *showed similar significant associations in females because all SNPs in the *MAOA *region show high linkage disequilibrium with each other (see Additional file 1). Common combinations of tag SNPs of *MAOA *were analyzed with the maximum post-operative pain ratings and showed similar tendency with the individual SNPs, as expected. However, this association is non-significant, even without the multiple testing corrections. Though *MAOA *and *MAOB *are closely located on the X chromosome, they have many different characteristics including the expression sites and substrates. In pain sensitivity, they also have different effects as *MAOB *SNPs do not show any significant association.

Since the re-uptake of monoamine neurotransmitters through specific transporters is the major method of elimination from the neural synapse, it is not surprising that the genetic variations in norepinephrine transporter gene (*SLC6A2*) and the serotonin transporter gene (*SLC6A4*) affect monoamine neurotransmitter mediated human behaviors including responses to painful stimuli. Unlike catabolizing enzyme encoding genes such as *COMT *and *MAO*, SNPs of transporter genes showed association with time dependant pain responses though it is not clear how these genetic variations influence pain or analgesic onset. Given the significant findings between genetic variations of monoamine neurotransmitter systems and sensitivity to clinical pain, additional high density genotyping of these genes regions may be informative. More than 30 SNPs with heterozygosity greater than 0.25 are reported and 6 nonsynonymous SNPs exist in *COMT *alone. Some of these SNPs induce nonsynonymous amino acid change though their heterozygosity is low or not known. Future studies including higher density genotyping around the candidate regions are needed.

Among the dopaminergic receptors, D2 receptor (DRD2) appears to be the major autoreceptor for dopaminergic neurons [39]. It was reported that DRD2 influences baseline nociception in the mouse, although this effect is weak and submodality selective. DRD2 receptors may contribute to attenuation of referred hypersensitivity caused by sustained nociception [40]. Despite the important role of DRD2 in the dopamine system, no significant association between genetic variations in *DRD2 *and clinically induced pain was found in our sample. Due to complicated interactions not only within monoamine neurotransmitter systems but also with related other enzymes and other pain modulating systems, it is not clear whether genetic polymorphisms in *DRD2 *affect pain sensitivity. Many association studies for common diseases suggest that many different genes distributed throughout the human genome contribute to the total genetic variability of a particular complex trait, with any single gene accounting for no more than a few percent of the overall variability of the trait [41].

Even though we found some significant association between genetic variations of monoamine neurotransmitter system and clinical pain sensitivity, for modest genetic effects and for identification of genotypic subgroups, much larger sample sizes are required. The SNPs showing association with pain sensitivity are in introns or untranslated regions. More complicated unidentified mechanism rather than the simple amino acid change of coding sequence may play a major role. Regardless of the significant associations, the risk of chance finding should be considered carefully. When we performed correction of the multiple testing with Bonferroni's correction, all of our significant associations were statistically non-significant. It should be emphasized that our data set is larger and more homogeneous than the studies reporting positive findings of *COMT *and pain [8,9]. Therefore, it is possible that the reported genetic associations between monoamine neurotransmitter system including metabolizing enzymes and transporters and pain are not justified by the available evidence.

A sample drawn from only one gender group may increase the ability to study phenotype-genotype association. It is reported that a gender difference exists in the genetic variation effect on pain responses [12,13,22,42,43]. However, it is still debatable that the independent analysis of each gender is necessary. It was reported that there is no evidence for a sex-specific genetic influence in the liability of heritability of clinical neck pain, especially in the clinical condition [44]. Analyzing females and males together in our study can be meaningful because overwhelming clinical pain and allelic frequencies are not different between genders as well as it enables us to compare the results of *COMT *with previously studies of Zubieta et al and Diatchenko et al. It is also necessary to take account of substructure of examined population even in a relatively homogenous genetic group such as the Icelanders. Even Icelanders cannot be considered to be a single, randomly interbreeding population [45]. This will probably be more important in larger populations like European Americans or African Americans. Descriptors such as ethnicity capture only some of the ancestral information about the biological and environmental factors that influence phenotypic characteristics [16]. Other potential interacting factors such as psychological profiles should also be considered in the future studies.

## Conclusion

Our results suggest that genetic polymorphisms in the monoamine neurotransmitter systems including *COMT*, *MAOA*, *SLC6A2 *and *SLC6A4 *may contribute but very weakly, if any, to the interindividual variability in pain responses to clinically induced acute injury. These results suggest that the previously reported associations between genetic polymorphisms in the monoamine neurotransmitter systems and the interindividual variability in pain responses cannot be replicated in a clinically relevant pain phenotype.

## Methods

### Subjects

The study was approved by the Institutional Review Board of the National Institute of Dental and Craniofacial Research and informed consent was obtained from all subjects. Among 221 patients undergoing oral surgery, European Americans (60 females and 52 males), age ranged from 17 to 35 years, were analyzed in this study. Patients underwent standardized surgery by the same oral surgeon removing third molar teeth that included at least one bony impacted mandibular third molar. After receiving pre-medication with intravenous midazolam (4.9 ± 0.2 mg) and local anesthesia with 2% lidocaine (250.6 ± 43.0 mg) with epinephrine 1:100,000, a mucoperiosteal flap was raised and retracted, bone removed, and the teeth were sectioned as needed to facilitate extraction of the impacted lower third molars.

### SNP genotyping

For genotyping, 50 ml of venous blood from each subject was collected. DNA isolation was performed with the Puregene™ DNA isolation kit (Gentra Systems Inc., Minneapolis, Minnesota, USA) following manufacturer's instructions.

For SNP genotyping, Assays-on-Demand or Assays-by-Design SNP Genotyping Products (Applied Biosystems, Foster City, California, USA) were used. Each well contained 2.5 μl of Taqman universal master mix, 0.25 μl of genotyping assay mix and 2.25 μl of DNAse free water. Polymerase chain reaction (PCR) was performed under the following conditions: 95 °C, 10 min followed by 40 cycles of 92 °C, 15 seconds and 60 °C, 1 minute in a Perkin-Elmer™ 9700 thermocycler (Perkin-Elmer Inc., Boston, Massachusetts, USA). Following PCR, fluorescence of each well was measured using the ABI Prism 7900 Sequence Detection System (Applied Biosystems, Foster City, California, USA). From the genomic sequences including their flanking regions, 46 SNPs from *COMT, MAOA, MAOB, SLC6A2, SLC6A3, SLC6A4 *and *DRD2 *were genotyped. Detailed information of genotyped SNPs is in Figure 1 and Table 1.

Genotype discrimination was performed using Taqman Sequence Detector version 2.1 software. Samples that failed to amplify were not included in the final analysis.

### Clinical pain measurement

Clinically induced pain was recorded with a paper and pencil form of a 100 mm visual analogue scale (VAS). After the extraction of the impacted third molars, pain was recorded every 20 minutes by VAS until subjects requested analgesic medication as the local anesthesia was eliminated and post-operative pain onset occurred. Ketorolac tromethamine (Toradol) was administered intravenously at the recommended dose (30 mg) and pain was recorded by VAS again at 15 minutes interval for 180 minutes. The maximum post-operative pain rating, onset time of post-operative pain, and the analgesic onset time after medication were used as measures of clinical pain.

### Data analysis

Analysis of variance (ANOVA) with Duncan's post hoc analysis was used to examine the effects of individual SNPs on pain response. A probability of < 0.05 was considered to be significant for statistical comparison. The total number of statistical comparisons evaluating the association between genetic variation and pain responses in this study was 138.

## Competing interests

The author(s) declare that they have no competing interests.

## Authors' contributions

HK carried out the molecular genetic studies, participated in the DNA extraction, genotyping, statistical analysis and drafted the manuscript. HL carried out the genotyping. JR participated in the collection of clinical pain data and surgery procedure. JB participated in the oral surgery. RD conceived of the study and participated in its design and coordination and helped to draft the manuscript. All authors read and approved the final manuscript.

## Supplementary Material

Additional File 1 Table of D' and r^2 ^matrices of *MAOA *in European American females Click here for file
