# Supplementary material for: Genetic polymorphisms in monoamine neurotransmitter systems show only weak association with acute post-surgical pain in humans
Source: Mol Pain. 2006 Jul 18;2:24. doi: 10.1186/1744-8069-2-24 (PMC1543620; doi:10.1186/1744-8069-2-24)
Supplement: Additional File 1 — Table of D' and r2 matrices of MAOA in European American females [file 1744-8069-2-24-S1.doc]

D´ and r^2^ matrices of *MAOA* in European American females

L1 L2 D' LOD r^2 CIlow CIhi Dist T-int

snp1 snp2 0.983 61.92 0.852 0.92 1.0 35838 277.86

snp1 snp3 0.983 61.92 0.852 0.92 1.0 42212 -

snp1 snp4 0.934 56.56 0.805 0.87 0.97 56805 -

snp1 snp5 0.869 49.63 0.731 0.79 0.92 83778 -

snp1 snp6 0.868 47.83 0.706 0.79 0.92 83999 -

snp1 snp7 0.869 49.63 0.731 0.79 0.92 86027 -

snp1 snp8 0.901 52.46 0.761 0.83 0.95 91318 -

snp2 snp3 1.0 80.66 1.0 0.97 1.0 6374 520.52

snp2 snp4 0.856 48.07 0.7 0.78 0.91 20967 -

snp2 snp5 0.835 42.66 0.634 0.75 0.9 47940 -

snp2 snp6 0.806 40.9 0.611 0.72 0.87 48161 -

snp2 snp7 0.835 42.66 0.634 0.75 0.9 50189 -

snp2 snp8 0.839 44.83 0.661 0.76 0.9 55480 -

snp3 snp4 0.856 48.07 0.7 0.78 0.91 14593 694.35

snp3 snp5 0.835 42.66 0.634 0.75 0.9 41566 -

snp3 snp6 0.806 40.9 0.611 0.72 0.87 41787 -

snp3 snp7 0.835 42.66 0.634 0.75 0.9 43815 -

snp3 snp8 0.839 44.83 0.661 0.76 0.9 49106 -

snp4 snp5 0.951 61.91 0.863 0.89 0.99 26973 786.75

snp4 snp6 0.936 62.06 0.863 0.88 0.97 27194 -

snp4 snp7 0.951 61.91 0.863 0.89 0.99 29222 -

snp4 snp8 0.92 59.22 0.834 0.86 0.96 34513 -

snp5 snp6 1.0 74.46 0.969 0.96 1.0 221 817.46

snp5 snp7 1.0 78.65 1.0 0.97 1.0 2249 -

snp5 snp8 1.0 74.46 0.969 0.96 1.0 7540 -

snp6 snp7 1.0 74.46 0.969 0.96 1.0 2028 593.97

snp6 snp8 0.968 70.29 0.938 0.92 0.99 7319 -

snp7 snp8 1.0 74.46 0.969 0.96 1.0 5291 323.26
